# Impact of COVID-19 on endoscopic follow-up of gastroesophageal varices

**DOI:** 10.1186/s43066-022-00223-x

**Published:** 2022-11-29

**Authors:** Amr M. Elsayed, Yasser M. Fouad, Hatem A. Hassan, Taha M. Hassanin, Abbas M. Abbas, Alshymaa A. Hassnine

**Affiliations:** 1 Department of Tropical Medicine and Gastroenterology, Faculty of Medicine, Minia University, Minia, Egypt; 2 Department of Internal Medicine, Faculty of Medicine, Minia University, Minia, Egypt; 3 Department of Biochemistry and Molecular Biology, Faculty of Medicine, Cairo University, Cairo, Egypt

**Keywords:** COVID-19, Portal hypertension, Gastroesophageal varices, Variceal ligation, Gastrointestinal bleeding, Endoscopy, Eradication, Follow-up

## Abstract

**Background:**

Portal hypertension is considered as a major complication of liver cirrhosis. Endoscopy plays a main role in managing of gastrointestinal complications of portal hypertension. Endoscopists are at increased risk for COVID-19 infection because upper gastrointestinal (GI) endoscopy is a high-risk aerosol-generating procedure and may be a potential route for COVID-19.

**Objectives:**

To compare the outcome between cirrhotic patients who underwent classic regular endoscopic variceal ligation after primary bleeding episode every 2–4 weeks, and those presented during the era of COVID-19 and their follow-up were postponed 2 months later.

**Methods:**

This retrospective study included cross-matched 238 cirrhotic patients with portal hypertension presented with upper GI bleeding, 112 cirrhotic patients presented during the era of COVID19 (group A) underwent endoscopic variceal ligation, another session after 2 weeks and their subsequent follow-up was postponed 2 months later, and 126 cirrhotic patients as control (group B) underwent regular endoscopic variceal band ligation after primary bleeding episode every 2–4 weeks.

**Results:**

Eradication of varices was achieved in 32% of cases in group A, and 46% in group was not any statistically significant (*p* > 0.05); also, there was no any statistical significant difference between both groups regarding occurrence of rebleeding, post endoscopic symptoms, and mortality rate (*p* > 0.05).

**Conclusion:**

Band ligation and injection of esophageal and gastric vary every 2 months were as effective and safe as doing it every 2 to 4 weeks after primary bleeding episode for further studies and validation.

## Introduction

Portal hypertension is considered as a major sequelae of liver cirrhosis, and its manifestations including ascites, gastroesophageal variceal hemorrhage, encephalopathy, and hepatorenal syndrome lead to increased morbidity and mortality [1].

Portal hypertension can be defined as rising in the portal venous system pressure. Pressure in the portal vein ranges normally from 7 to 12 mm Hg at rest and in fasting conditions [2]. Portal hypertension happens due to rise in resistance or blood flow in the porto splenic venous system, so in liver cirrhosis, the formation of fibrous tissue scar and cirrhotic nodules can lead to an increase in intrahepatic vascular resistance and consequently portal pressure [3]. Portal hypertension leads to an increase in the portosystemic collateral flow to decompress the portal venous system. The most clinically important site of these collaterals is within the mucosa of the proximal stomach and distal esophagus, resulting in the development of gastroesophageal varices [4].

Screening for gastroesophageal varices with upper gastrointestinal endoscopy has been recommended for all patients with cirrhosis. The value of early detection of varices lies in the hazard of their rupture and subsequently horrible bleeding. Esophageal varices can be classified as small (< 5 mm) or large (5 mm). The risk of bleeding in small varices is approximately 5% per year and up to 15% in large varices [5].

A new acute severe respiratory syndrome coronavirus outbreak leading to coronavirus disease 2019 (COVID-19) has started in Wuhan since December 2019 and then rapidly spread and became worldwide [6]. The World Health Organization classified COVID-19 as pandemic infectious disease; this was on 11 March 2020, and the number of confirmed COVID-19 cases had increased to more than 37,2000 globally by 24 March 2020 [7].

Gastrointestinal endoscopies, especially those done through the nasal and oral routes, might lead to upper respiratory symptoms such as cough and subsequent dissemination of droplets and increase the risk of exposure of the medical team, including endoscopists, anesthetists, nurses, and assistants to aerosol contamination [8]. The hazard of viral transmission may increase during a prolonged stay in a closed environment as in endoscopic room [9].

GI endoscopy units are faced with great dares during this pandemic as 3.8% of established cases from China were healthcare personnel (HCP) with reported deaths [10]. GI endoscopy is likely a hazardous procedure as pulmonary and gastric secretions, as well as fecal material, may contain large viral loads. Infection control measures must be applied to ensure patient safety, avoid nosocomial outbreaks, safe heathcare personnel, and ensure rational use of limited personal protective equipment (PPE) [11]. In this study, we aim to compare the outcome between cirrhotic patients who underwent classic regular endoscopic variceal ligation after primary bleeding episode every 2–4 weeks, and those presented during the era of COVID-19 and their follow-up was postponed 2 months later.

## Subjects and methods

This retrospective study included 238 cirrhotic patients with portal hypertension presented with upper GIT bleeding to GIT endoscopy unit Minia University Hospital. One-hundred twelve cirrhotic patients (group A) presented to the hospital in 2020 during the 1st era of COVID-19 from April to July 2020 underwent endoscopic variceal ligation (EVBL) and gastric varices injection if needed another session after 2 weeks, and their subsequent follow-up was postponed 2 months later. This was done according to WHO and Egyptian Ministry of Health recommendations to postpone elective maneuvers including the elective endoscopic sessions to minimize COVID-19 transmission. Another cross-matched 126 cirrhotic patients (group B) were hospitalized in 2019 and underwent regular endoscopic variceal band ligation and gastric varices injection if needed after primary bleeding episode every 2–4 weeks. Patients with hepatic encephalopathy, patients with portal vein thrombosis, patients with contraindications to use beta-blockers following EVBL, and patients with a platelet count less than 50,000/mm^3^ or INR more than 1.8 are not included in the study. All patients were assessed clinically by detailed history and meticulous examination. Complete blood count, ALT, AST, serum albumin, serum bilirubin, serum creatinine, INR, and abdominal ultrasonography were done to all patients. We used Child-Turcotte-Pugh classification and model for end-stage liver disease (MELD) score to evaluate these cirrhotic patients [12, 13]. The endoscopic maneuver was performed in a single endoscopy unit using video-scopes Pentax EG 2990 I and EG2990 K and using light-source EPK-I 5000 by experienced gastroenterologists. Patients were sedated with intravenous midazolam. A gastroscopy was done, and if medium to large size esophageal varices were present, then bands were applied by using multi-load ligature device. Patients were observed for 1 h following the procedure and discharged with clear instructions. Grading of esophageal varices was performed as small varices if they were < 5 mm large and if they were > 5 mm [14, 15]. Bleeding gastric varices are injected with cyanoacrylate and lipidol [16].

The collected data were tabulated and statistically analyzed using the Statistical Package for Social Sciences (SPSS) program software version 24. Qualitative data were expressed as proportions, quantitative data is expressed in the form of mean + standard deviation (SD) and median plus interquartile range (IQR). Statistical significance was defined if *p*-values are less than or equal to 0.05.

## Results

The present retrospective study included cross-matched 238 cirrhotic patients who were suffering portal hypertension and presented with upper GIT bleeding; 112 ones presented during the 1st era of COVID-19 from April to July 2020, and their subsequent follow-up endoscopic sessions were 2 months later (group A), and another 126 cirrhotic patients were presented during 2019 and had subsequent regular endoscopic follow-up (group B). Table 1 showed the baseline characteristics of the studied groups at the time of presentation by bleeding to the hospital; males were more predominant than females; most patients in both groups is presented with hematemesis and melena; the majority of patients were child class B: 59% of group A and 62% of group B; the median of MELD score in group A was 12 and 11 in group B; and the median number of esophageal variceal cords in group A was 4 and 4 cords in group B. Majority of patients had large varices: 69% in group A and 66% in group B. Table 2 showed that eradication of varices was achieved in 36 cases (32%) in group A and 58 patients (46%) in group B without significant difference (*P* > 0.05). Rebleeding occurred in 18 patients (16%) in group A and 32 ones (25%) in group B without statistical difference (*p* > 0.05). Dysphagia, odynophagia, and chest pain were different post endoscopic symptoms occurring in 22 patients (20%) in group A and in 28 patients (22%) in group B without any significant statistical difference (*p* > 0.05). Eight patients died in group A (7%), while 14 patients died in group B (11%) without significant statistical difference between them (*p* > 0.05).

**Table 1:** The baseline characters of the studied groups

	Group A (112)	Group B (126)
Age (range in years)	46 (17–71)	48 (21–66)
Female/male	44/68	52/74
Hematemesis	16 (14%)	28 (22%)
Melena	18 (16%)	22 (18%)
Both	78 (70%)	76 (60%)
Child class (number & %)		
A	21 (19%)	26 (21%)
B	66 (59%)	78 (62%)
C	25 (22%)	22 (17%)
Hemodynamic instability number & %	51 (49%)	62 (55%)
Median MELD score	12 (7–34)	11 (8–33)
INR	1.5 (1.1–2.4)	1.6 (1.2–2.6)
Bilirubin (mg/dl)	1.3 (0.9–14)	1.5 (1–21)
Albumin (g/dl)	3.2 (2.4–4.1)	3 (2.2–4.5)
Creatinine (mg/dl)	1 (0.9–2.8)	0.9 (0.8–6.2)
ALT	48 (26–212)	44 (18–466)
AST	52 (22–288)	49 (16–512)
Median number of varices	4 (1–6)	4 (1–6)
Size of varices		
Small	34 (31%)	42 (34%)
Large	78 (69%)	84 (66%)

**Table 2:** Outcome of endoscopic intervention

Eradication rate	36/112 (32%)	58/126 (46%)	*p* = 0.145
Rebleeding rate	18/112 (16%)	32/126 (25%)	*p* = 0.07
Dysphagia, odynophagia, chest pain	22/112 (20%)	28/126 (22%)	*p* = 0.62
Death	8/112 (7%)	14/126 (11%)	*p* = 0.194

## Discussion

The present study is designed to assess increasing duration of sequential follow-up endoscopic sessions of esophagogastric varices every 2 months during the 1st era of COVID-19 in comparison with the classic way of endoscopic follow-up every 2 to 4 weeks before the era of the COVID in 2019. The pandemic COVID-19 has made a lot of changes in medical practice. Now patients and physicians were struggling to reduce classic visits for public health reasons [17]. In our study, we have found that eradication of esophagogastric varices in those followed up during era of COVID-19 in 2020 was not significantly different from those followed up endoscopically every 2 to 4 weeks during the same period in 2019. Surprisingly, we also found that there is not any significant difference between both groups of patients regarding the occurrence of rebleeding. There was no significant statistical difference regarding the occurrence of post endoscopic symptoms like dysphagia, odynophagia, or chest pain. The mortality rate in patients who underwent endoscopic follow-up every 2 months was not significantly different from those who underwent regular classic follow-up every 2 to 4 weeks.

## Conclusion

During our endoscopic practice in the era of COVID-19, we can conclude that band ligation of esophageal varices and injection of gastric varices every 2 months seem to be as effective and safe as doing it every 2 to 4 weeks after primary bleeding episode for more studies.

## Data Availability

This published article contains all the knowledge produced or analyzed during this research.
